# ASO Author Reflections: From Polypharmacy to Procedure—Defining Value in the Management of Primary Aldosteronism

**DOI:** 10.1245/s10434-026-19562-9

**Published:** 2026-04-02

**Authors:** Jesse E. Passman, Heather Wachtel

**Affiliations:** 1 Department of Surgery, University of Pennsylvania Health System, Philadelphia, USA; 2 University of Pennsylvania Perelman School of Medicine, Philadelphia, USA

## Past

Despite its prevalence as the leading driver of secondary hypertension, primary aldosteronism (PA) remains a frequently overlooked clinical entity. For patients presenting with unilateral disease, adrenalectomy offers rates of biochemical cure approaching 100%, as well as significant improvements in blood pressure control, cardiovascular outcomes, and prevention of comorbid disease.^1,2^ While the clinical benefits of adrenalectomy in this population are well-documented, much of the literature has focused on biochemical end points, blood pressure normalization, and long-term survival. Comparatively little attention has been directed toward the tangible day-to-day consequences of treatment strategy, particularly antihypertensive medication burden and prescription costs. Although simulation and Markov modeling studies suggest that surgery can be cost-effective, real-world data directly comparing medication trajectories and associated costs between surgical and medical management strategies have been limited. Medication burden and downstream costs represent critical outcomes for both patients and healthcare systems which must be characterized to inform best practices.

## Present

Using a large national administrative claims database, we compared antihypertensive medication use and prescription costs among patients with newly diagnosed PA who were subsequently managed with adrenalectomy or medical therapy. Both cohorts demonstrated similar antihypertensive medication burden and costs at baseline. Over the following year, however, those who underwent adrenalectomy were prescribed significantly fewer antihypertensive medications; adrenalectomy was associated with a drop in antihypertensive prescriptions from 2.9 to 1.5 medications, whereas medically managed patients saw their antihypertensive burden drop from 2.8 to 2.5 medications. Surgical management was also associated with substantial reductions in cumulative antihypertensive prescription costs ($908 over the first year) and marked reduction in need for potassium supplementation. Notably, these benefits were observed even among high-risk patients with resistant hypertension, a population often perceived as less likely to achieve medication independence.^3^

These findings reinforce that the benefits of adrenalectomy in primary aldosteronism extend beyond traditional end points of biochemical cure. Surgical management meaningfully improves medication trajectories within the first year, translating to measurable cost savings. Despite the upfront procedural cost of adrenalectomy, about $26,800 in our cohort, the downstream reduction in medication burden represents an early and tangible return on that investment. This reduced antihypertensive medication burden in turn likely reflects improved physiological control of disease, with anticipated long-term benefits regarding comorbid cardiovascular risk, medication adherence, and quality of life.

## Future

The management of PA exemplifies a broader principle in surgical oncology and endocrine surgery: definitive surgical therapy may entail upfront costs but can substantially reduce chronic treatment burden. As healthcare systems shift toward value-based frameworks, understanding how surgical intervention reshapes long-term resource utilization is essential. Future work should extend beyond the 1-year time horizon to quantify lifetime costs savings not only for medication costs, but also for cumulative healthcare utilization to account for diminished accumulation of comorbidities. Incorporation of cardiovascular event rates, quality-of-life metrics, and productivity measures will provide a more comprehensive assessment of value.

Ultimately, the benefits of surgical management cannot be realized, however, unless patients with PA are identified via innovative, expansive screening programs. Despite increasing awareness, screening rates for PA remain disappointingly low. It is critical that future work emphasize the actual implementation of established best practices across health systems, such as via the use of electronic health record-based clinician nudges.^4,5^ Patient navigation programs should be considered to equitably facilitate timely transition along the complex care pipeline required to go from positive screen to definitive management. Facilitation of care across the continuum of screening, diagnosis, and management is essential for the theoretical benefits of surgical management to be realized at the system level.
